# Calreticulin couples with immune checkpoints in pancreatic cancer

**DOI:** 10.1002/ctm2.10

**Published:** 2020-04-18

**Authors:** Xing Huang, Tianyu Tang, Xun Wang, Xueli Bai, Tingbo Liang

**Affiliations:** ^1^ Zhejiang Provincial Key Laboratory of Pancreatic Disease The First Affiliated Hospital School of Medicine Zhejiang University Zhejiang China; ^2^ Department of Hepatobiliary and Pancreatic Surgery The First Affiliated Hospital School of Medicine Zhejiang University Zhejiang China; ^3^ Innovation Center for the Study of Pancreatic Diseases Zhejiang China

**Keywords:** calreticulin, cancer immunotherapy, immune checkpoint, immunogenic cell death, immunogenic chemotherapy, pancreatic cancer

## Abstract

Although immune checkpoint blockade is considered to be the dominant approach in future cancer immunotherapy, whether it will apply to pancreatic cancer remains largely unknown. To address this issue, pancreatic cancer–associated datasets were individually collected by Gene Expression Profiling Interactive Analysis 2 (GEPIA2), cBioPortal, and Tumor and Immune System Interaction Database (TISIDB), and subsequently subjected to prognostic, genomic, and immunologic analyses of all well‐established immune checkpoints. The results indicate that immune checkpoints might not be ideal targets for pancreatic cancer therapy. Intriguingly, the genomic alteration of calreticulin, the key mediator of chemotherapy‐induced cancer immunogenic cell death, was found to couple with immune checkpoints in pancreatic cancer. Moreover, calreticulin was observed to be highly expressed in pancreatic adenocarcinoma, and high calreticulin expression significantly favors both overall survival and disease‐free survival of patients with pancreatic adenocarcinoma. Importantly, calreticulin was further revealed to be closely related to anti‐tumor immunity in pancreatic adenocarcinoma, including multiple immune effector molecules and T‐cell signatures. Taken together, calreticulin‐based therapy may represent a more promising prospect for pancreatic cancer immunotherapy than immune checkpoint blockade therapy.

**Abbreviations**
ANXA1: annexin A1
CALR: calreticulin
DFS: disease‐free survival
HR: hazard ratio
HMGB1: high‐mobility group box 1
IFNAR1: type I interferon receptor 1
OS: overall survival
PAAD: pancreatic adenocarcinoma
PANX1: pannexin 1

## 1 INTRODUCTION

Increasing evidence suggests that among the most promising therapies for cancer, immune resistance is the immune checkpoint blockade.^1^ To date, multiple immune checkpoints have been identified, at least on a functional level if not yet mechanistically, including but not limited to PD‐L1 (CD274), PD‐L2 (PDCD1LG2), CD80, CD86, VTCN1, VSIR, HHLA2, TNFRSF14, PVR, CD112 (NECTIN2), CD200, LGALS9, ICOSLG, TNFSF9, TNFSF4, CD70, TNFSF18, and CD48.^2^, ^3^ However, whether and how such immune checkpoints are involved in the prognosis and therapeutic efficacy of pancreatic cancer remain largely unclear. Recently, advanced technology including genomic investigation has become the most efficient method of accelerating clinical and translational cancer research and therapy.^4^, ^5^, ^6^, ^7^ Previous research has shown that immune checkpoints and some other gene‐expression patterns are closely correlated with disease prognosis of several specific carcinomas, and can also be used to predict the ideal cancer types that will benefit from treatment with cancer immunotherapy.^8^, ^9^, ^10^ Therefore, this study aimed to clarify the clinical significance of immune checkpoints in pancreatic cancer patients, and propose more suitable strategies of improving antipancreatic cancer immunity using a high‐throughput sequencing database.

## 2 MATERIALS AND METHODS

To analyze the immune checkpoint‐related prognosis in pancreatic cancer, pancreatic cancer genomics related datasets in TCGA (http://cancergenome.nih.gov), International Cancer Genome Consortium (ICGC, https://icgc.org), and other open access databases were individually collected, and subsequently subjected to a bioinformatics analysis by web servers, Gene Expression Profiling Interactive Analysis 2 (GEPIA2, http://gepia2.cancer-pku.cn), cBioPortal for Cancer Genomics (http://www.cbioportal.org), and Tumor and Immune System Interaction Database (TISIDB, http://cis.hku.hk/TISIDB), respectively.^22^, ^23^, ^24^, ^25^, ^26^ Briefly, GEPIA2 was used to calculate the prognostic indexes, including the differential expression, pathological stage, gene correlation, and patient survival; cBioPortal was used to conduct visualization and comparison of gene alterations; TISIDB was used to explore the correlation between abundance of immunomodulators and expression of inquired gene. In detail, one‐way analysis of variance method was used for differential analysis of gene expression, and genes with higher |log2FC| values (>1) and lower *Q*‐values (<0.01) were considered differentially expressed genes. log2(TPM+1) was used for log‐scaling differential expression in different pathological stages, and a Pr(>F) < 0.05 was considered to be statistically significant. OS and DFS analyses were performed using the Kaplan‐Meier method with 50% cutoff for both low‐ and high‐expression groups. Log‐rank test, also known as the Mantel‐Cox test, was used for hypothesis test. The Cox proportional hazard ratio (HR) and the 95% confidence interval information were also included in the survival plots. A *P*‐value < 0.05 was considered to be statistically significant. Spearman method was used to analyze the pair‐wise gene expression correlations, and a *P*‐value < 0.05 was considered statistically significant. The correlated degree was identified by the absolute value of the correlation coefficient: ≤0.4, weak; 0.41‐0.60, moderate; 0.61‐0.80, strong; and 0.81‐1.0, very strong. The co‐occurrence and mutual exclusivity of genetic alteration between inquired gene and each immune checkpoint was determined by log2 odds ratio, *P*‐value, and *Q*‐value. A *Q*‐value < 0.05 was considered to be statistically significant. The investigated immunoinhibitors were collected according to Charoentong's study, and each Spearman correlation between inquired gene and a distinct immunoinhibitor in an individual cancer type was integrated into the indicated heatmap.

## 3 RESULTS

**FIGURE 1:**
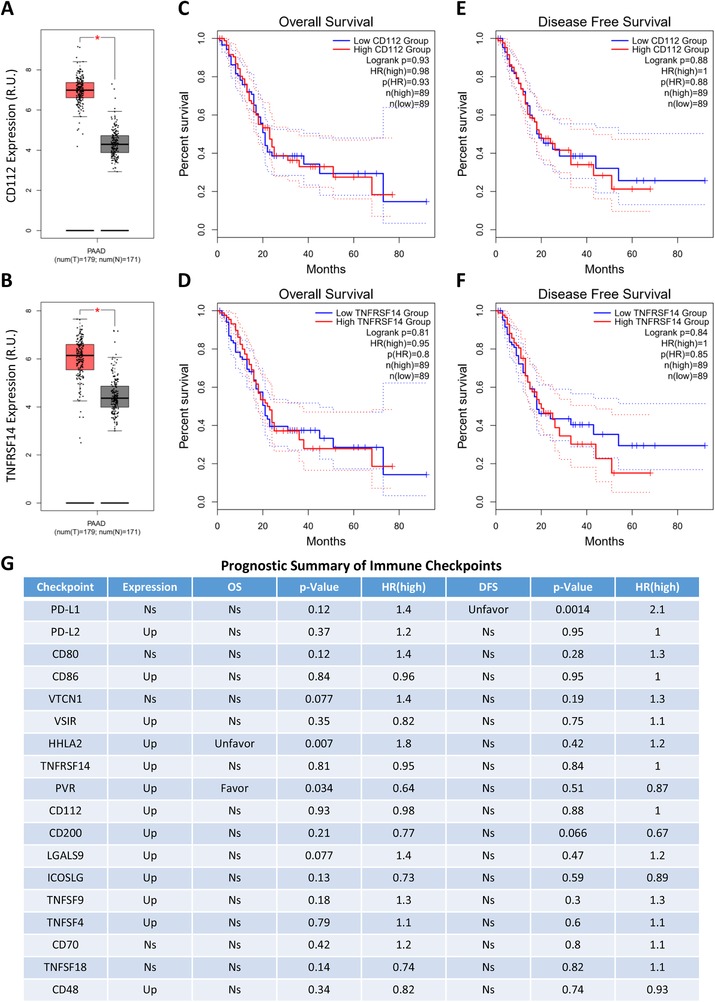
Prognostic analysis of immune checkpoints in pancreatic cancer. A and B, Differential expression analysis of representative immune checkpoints in pancreatic cancer. GEPIA generates box plots with jitter (size = 0.4) for comparing CD112 (A) and TNFRSF14 (B) expression in pancreatic cancer and paired normal tissues (TCGA tumor versus TCGA normal + GTEx normal). Peach cluster: tumor samples; gray cluster: normal samples. The method for differential analysis is one‐way analysis of variance (ANOVA). Genes with higher |log2FC| values (>1) and lower *Q*‐values (<0.01) were considered differentially expressed genes. C and D, Overall survival (OS) analysis of representative immune checkpoints in pancreatic cancer. GEPIA generates Kaplan‐Meier OS curves comparing the groups with different expression levels of CD112 (C) and TNFRSF14 (D) in pancreatic cancer (TCGA tumor). E and F, Disease‐free survival (DFS) analysis of representative immune checkpoints in pancreatic cancer. GEPIA generates Kaplan‐Meier DFS curves comparing the groups with different expression levels of CD112 (E) and TNFRSF14 (F) in pancreatic cancer (TCGA tumor). Blue line: low‐expression groups (50% cutoff); red line: high‐expression groups (50% cutoff). Log‐rank test, also known as the Mantel‐Cox test, was used for hypothesis test. The Cox proportional hazard ratio and the 95% confidence interval information were also included in the survival plots. *P*‐value < .05 was considered to be statistically significant. G, Prognostic summary of the immune checkpoints in pancreatic cancer. The detailed differential expression profile, OS, DFS, *P*‐value, and HR (high) of all representative immune checkpoints were individually summarized as indicated. *P*‐value < .05 was considered to be statistically significant (Up: upregulated in tumor; Favor: favorable to survival; Unfavor: unfavorable to survival; Ns: nonsignificant)

Despite PD‐1/PD‐L1 and CTLA‐4 as the most important immune checkpoints, emerging evidence indicates CD112 and TNFRSF14 are also the representative ones.^11^ Differential expression and pathological stage analyses revealed that in comparison to the normal pancreas tissue, the expression levels of CD112 and TNFRSF14 were not significantly deregulated in the pancreatic tumors (Figure 1A and B), and did not regularly fluctuate between each of the two different stages (Figure S1A and B). As the mutations of KRAS and TP53 are considered to be the leading causes of pancreatic cancer, the relationship between CD112/TNFRSF14 and KRAS/TP53 was individually analyzed. The results showed that the correlations of CD112/TNFRSF14 and KRAS (Figure S1C and D), as well as CD112/TNFRSF14 and TP53 (Figure S1E and F), were actually quite weak and even nonsignificant in pancreatic cancer. Furthermore, the survival analyses of patients with pancreatic cancer clearly indicated that the expression levels of CD112 and TNFRSF14 had no significant influence on overall survival (OS) (Figure 1C and D) and disease‐free survival (DFS, also called relapse‐free survival [RFS]) (Figure 1E and F). These results indicated that the clinical significance of CD112 and TNFRSF14 is limited in pancreatic cancer. Moreover, there were no competing outcomes in the prognostic analyses for any of the representative immune checkpoints (Figure 1G). Briefly, the overall phenotypes could be categorized according to the three negative conditions: (a) without differential expression between pancreatic cancer and the normal samples (eg, PD‐L1 and CD80); (b) upregulated in pancreatic cancer but without any significant relevance to the OS and DFS of pancreatic cancer patients (eg, PD‐L2 and CD86); and (c) upregulated in pancreatic cancer, significantly related to the OS and DFS of pancreatic cancer patients (favorable or unfavorable), but with both stimulatory and inhibitory potential on immune system (eg, HHLA2 and PVR). Furthermore, the relatively weak and even nonsignificant correlations were observed between immune checkpoints and pathological stage or KRAS/TP53 expression, respectively (Figure S1G), which further implicated that immune checkpoints themselves might not be ideal targets for pancreatic cancer.

Chemotherapy is the dominant approach for pancreatic cancer therapy, at least at the current clinical stage. In addition to the direct killing effects on tumors, increasing evidence suggests the tumoral immunogenicity caused by chemotherapy‐induced specific cell death is more important for the therapeutic efficacy in an immune system–dependent manner. Several key factors have been identified that are involved in mediating immunogenic chemotherapy, including, but not limited to calreticulin (CALR), annexin A1 (ANXA1), high‐mobility group box 1 (HMGB1), type I interferon receptor 1 (IFNAR1), and pannexin 1 (PANX1).^12^, ^13^ Among these molecules, CALR and its exposure, indicating translocation from the endoplasmic reticulum to the cell membrane, play a dominant role in dictating chemotherapy‐induced cancer immunogenic cell death, and anticancer immune response in multiple cancers (eg, melanoma, sarcoma, colorectal carcinoma, and breast cancer).^14^, ^15^, ^16^ However, whether CALR is also indicative of therapeutic efficacy in pancreatic cancer remains largely unknown. Although Matsukuma et al previously reported that CALR expression was upregulated in stem‐like cells in pancreatic cancer, higher CALR protein expression was associated with poorer rather than better survival in pancreatic cancer patients. Of note, the cohort was relatively small, and performed at only a single center.^17^ Therefore, it is urgent to elucidate the potential role of CALR in antipancreatic cancer immunity.

**FIGURE 2:**
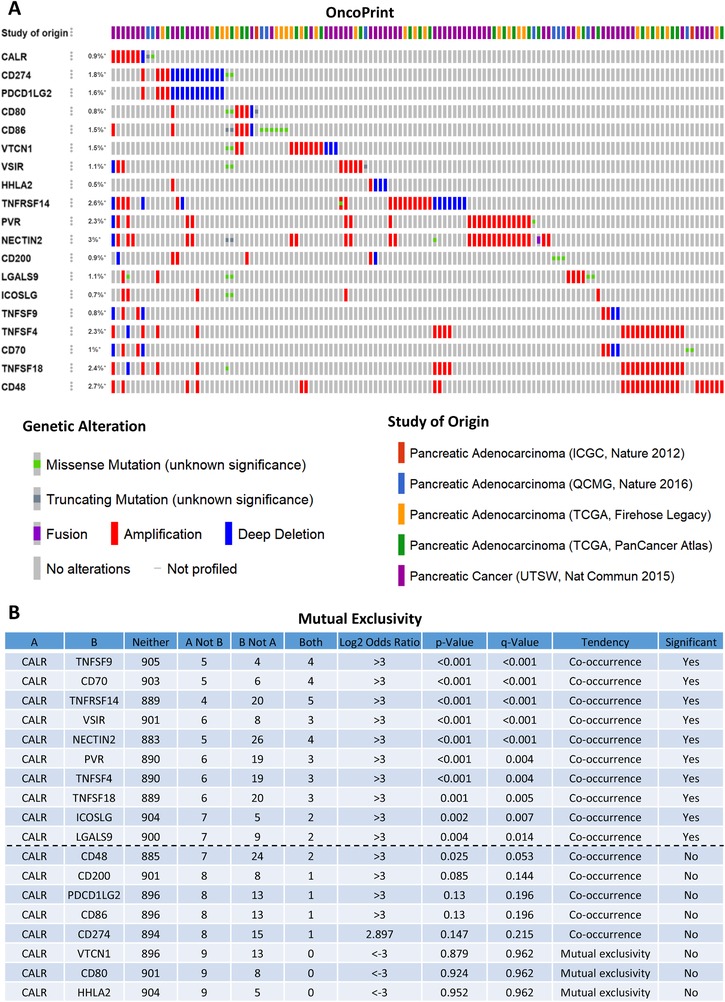
Immunogenic calreticulin (CALR) couples with immune checkpoints in pancreatic cancer. A, Landscape of CALR and immune checkpoint alteration in pancreatic cancer. Compact visualization of cases with multiple genetic alterations of CALR and immune checkpoints (origined from five studies) were individually shown by cBioPortal as indicated, including fusion, amplification, deep deletion, truncating mutation, and missense mutation. B, Mutual‐exclusivity analysis between CALR and multiple‐immune checkpoints in pancreatic cancer. The altered relationship between CALR and each immune checkpoint, such as co‐occurrence and mutual exclusivity, was presented as indicated. The detailed log2 odds ratio, *P*‐value, *Q*‐value, tendency, and significance were individually presented in each panel. *Q*‐value < 0.05 was considered to be statistically significant (indicated as yes, and others as no)

Intriguingly, the genomic investigation revealed that CALR was actually involved in the alteration of immune checkpoints in pancreatic cancer. The general landscape of CALR and immune checkpoint alteration in pancreatic cancer was compactly visualized, including fusion, amplification, deep deletion, truncating, and missense mutations (Figure 2A). The detailed relationship between CALR and each representative immune checkpoint was individually presented as indicated in Figure 2B. Of note, the CALR alteration showed a statistically significant co‐occurrence rather than mutual exclusivity with extensive immune checkpoints, such as TNFSF9, CD70, TNFRSF14, VSIR, NECTIN2, PVR, TNFSF4, TNFSF18, ICOSLG, and LGALS9. These findings strongly indicate that CALR is a potential coregulator of immune checkpoints in pancreatic cancer. Additionally, the genetic alteration of CALR also shows significant co‐occurrence with each of all the investigated immune checkpoints in prostate adenocarcinoma (Figure S2A and 2B). This similar even much stronger phenomenon observed in prostate adenocarcinoma suggested the potential universality of CALR‐immune checkpoint interplay, at least not merely in pancreatic cancer.

**FIGURE 3:**
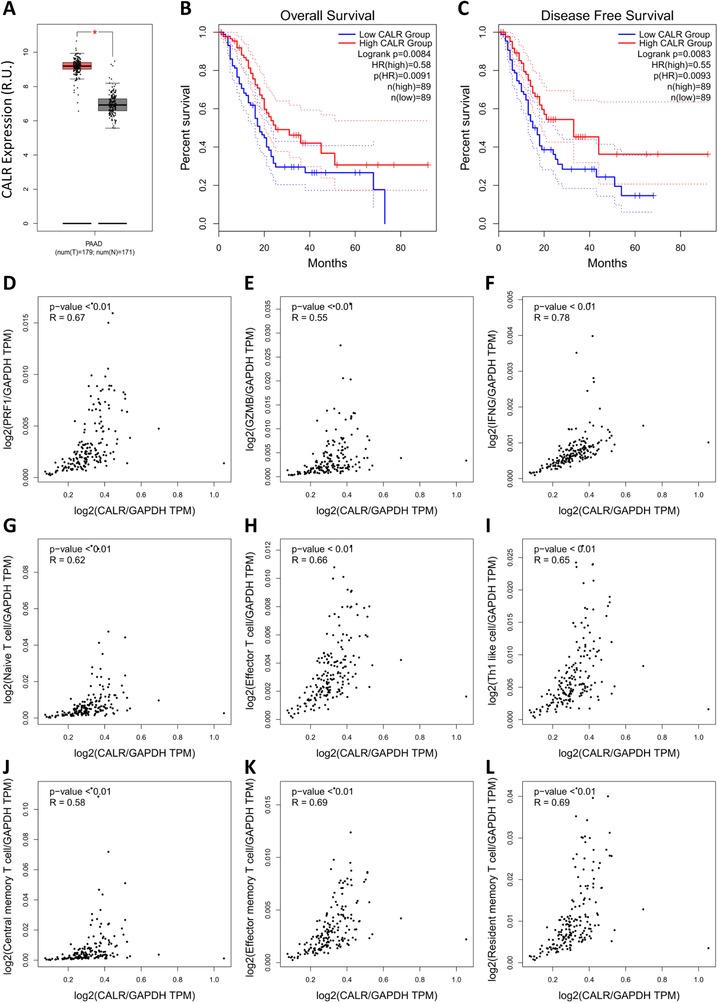
Prognostic analysis and immune correlation of CALR in pancreatic cancer. A, Differential expression analysis of CALR in pancreatic cancer. GEPIA generates box plot with jitter (size = 0.4) for comparing CALR expression in pancreatic cancer and paired normal tissues (TCGA tumor versus TCGA normal + GTEx normal). Peach cluster: tumor samples; gray cluster: normal samples. The method for differential analysis is one‐way ANOVA. Genes with higher |log2FC| values (>1) and lower *Q*‐values (<0.01) were considered differentially expressed genes. B, OS analysis of CALR in pancreatic cancer. C, DFS analysis of CALR in pancreatic cancer. GEPIA generates Kaplan‐Meier OS (B) and DFS (C) curves comparing the groups with different expression levels of CALR in pancreatic cancer (TCGA tumor). Blue line: low‐expression group (50% cutoff); red line: high‐expression group (50% cutoff). Log‐rank test, also known as the Mantel‐Cox test, was used for hypothesis test. The Cox proportional hazard ratio and the 95% confidence interval information were also included in the survival plots. *P*‐value < 0.05 was considered to be statistically significant. D, Correlation analysis of CALR and PRF1 in pancreatic cancer. E, Correlation analysis of CALR and GZMB in pancreatic cancer. F, Correlation analysis of CALR and IFNG in pancreatic cancer. G, Correlation analysis of CALR and naïve T‐cell signatures in pancreatic cancer. H, Correlation analysis of CALR and effector T‐cell signatures in pancreatic cancer. I, Correlation analysis of CALR and Th1‐like cell signatures in pancreatic cancer. J, Correlation analysis of CALR and central memory T‐cell signatures in pancreatic cancer. K, Correlation analysis of CALR and effector memory T‐cell signatures in pancreatic cancer. L, Correlation analysis of CALR and resident memory T‐cell signatures in pancreatic cancer. GEPIA generates the pair‐wise gene expression correlations of two genes, or between one gene and several signatures in pancreatic cancer (TCGA tumor) using Spearman method after normalization by GAPDH. The detailed *P*‐value and *R* were individually presented as indicated in each panel. *P*‐value < 0.05 was considered statistically significant

To evaluate the therapeutic potential of CALR‐based therapy in pancreatic cancer, the prognostic and immunologic correlation of CALR was further analyzed in detail. In contrast to the immune checkpoints, the results revealed that CALR is highly expressed in pancreatic cancer in comparison to the normal control tissue (Figure 3A). In addition, high CALR expression largely favored the OS and DFS of pancreatic cancer patients (Figure 3B and C). Meanwhile, prognosis of other representative regulatory factors of immunogenic chemotherapy was also analyzed. It was observed that although all of the expression levels of ANXA1, HMGB1, IFNAR1, and PANX1 were significantly upregulated in pancreatic cancer (Figure S3A‐D), the high expression of ANXA1, HMGB1, IFNAR1, and PANX1 was either nonsignificant or unfavorable for the OS (Figure S3E‐H) and DFS (Figure S3I‐L) of pancreatic cancer patients. These results further confirmed that among the key mediators of immunogenic chemotherapy, CALR is potentially the only one favorable for the survival of pancreatic cancer patients. Furthermore, CALR was significantly correlated with key immunity‐killing molecules, including PRF1 (Figure 3D), GZMB (Figure 3E), and IFNG (Figure 3F) in pancreatic cancer. Furthermore, CALR was found to be closely related to immune signatures, including the signaling patterns of naïve T cells (Figure 3G), effector T cells (CX3CR1/FGFBP2/FCGR3A) (Figure 3H), Th1‐like cells (CXCL13/HAVCR2/IFNG/CXCR3/BHLHE40/CD4) (Figure 3I), central memory T cells (CCR7/SELL/IL7R) (Figure 3J), effector memory T cells (PDCD1/DUSP4/GZMK/GZMA/IFNG) (Figure 3K), and resident memory T cells (CD69/ITGAE/CXCR6/MYADM) (Figure 3L). Together, this evidence strongly suggests that CALR had a crucial impact on the antitumor immune response in pancreatic cancer therapy.

Last but not the least, to better understand the potential roles and clinical relevance of CALR in multiple human cancers, the expression profiles of CALR were further investigated across 33 major types of human cancer in The Cancer Genome Atlas (TCGA) database. In comparison to the paried healthy tissues, CALR was expressed at higher levels in bladder urothelial carcinoma, breast invasive carcinoma, cervical squamous cell carcinoma and endocervical adenocarcinoma, colon adenocarcinoma, lymphoid neoplasm diffuse large B‐cell lymphoma, glioblastoma multiforme, brain lower grade glioma, liver hepatocellular carcinoma, ovarian serous cystadenocarcinoma, pancreatic adenocarcinoma (PAAD), prostate adenocarcinoma, rectum adenocarcinoma, skin cutaneous melanoma, stomach adenocarcinoma, testicular germ cell tumors, thymoma, uterine corpus endometrial carcinoma, and uterine carcinosarcoma (Figure S4A). More importantly, to deeply understand the association between CALR and immune regulation, the potential relevances of CALR to multiple cancer immunoinhibitors were further analyzed across 30 cancer types. It was observed that CALR expression levels correlated negatively with the relative abundance of major immunoinhibitors in several specific cancers, such as cholangio carcinoma, kidney chromophobe, lung adenocarcinoma, lung squamous cell carcinoma, PAAD, prostate adenocarcinoma, testicular germ cell tumors, and thyroid carcinoma (Figure S4B), suggesting these cancer types are the potentially optimal targets for CALR‐based immunotherapy.

Collectively, although immune checkpoint blockade has been highly successful in treating melanoma and several other cancers,^18^ the results of the prognosis and expression analyses in this study indicate that immune checkpoints may not be ideal therapeutic targets for pancreatic cancer. Indeed, the targeted capability and effectiveness of immune checkpoints depend on a number of influencing factors (eg, the degree of differential expression or mutation in neoplastic and normal tissues, the contribution to cancer patient survival, as well as the relativity to antitumor immunity).^19^ However, this does not mean that pancreatic cancer is not under the control of immune checkpoints. On the contrary, accumulating evidence suggests that pancreatic cancer likely coordinates several (but not single) immune checkpoints against immune attacks. Thus, individual targeting of each of immune checkpoints has variable efficacy in pancreatic cancer therapy, whereas combination treatment with drugs targeting all of the checkpoints may also be restricted due to potential side effects.

Fortunately, the rapid development of open high‐throughput sequencing databases due to advances in whole‐genome sequencing provides us with valuable information for investigating potential novel immune targets for treatment.^9^, ^10^ Recently, immune checkpoint blockade represents a breakthrough for cancer treatment, whereas immunogenic cell death, which includes CALR exposure, is also an important process in tumor immunotherapy. According to our results, immune checkpoint blockade therapy combined with radiotherapy and chemotherapy in CALR‐upregulated tumors might be a more appropriate therapeutic strategy for pancreatic cancer and provide patients with an extra survival benefit. It has been verified that a CALR alteration is closely associated with multiple immune checkpoints at a genomic level, which strongly implicates a possible co‐contribution to immune surveillance and evasion of pancreatic cancer. Thus, upregulating CALR rather than targeting immune checkpoints represents a potentially more efficient approach for pancreatic cancer therapy. Additionally, it should be emphasized that this study provides a foundation for further research, despite it being the first report describing CALR to be the genomic coupler of immune checkpoints. There are several emerging reports about the crucial roles of genomic correlation, biomarkers, transcriptional regulation, translational modulation, and posttranslational modification in immune checkpoint blockade.^4^, ^19^, ^20^, ^21^ Further exploration of the potentially direct interplay or indirect influence between CALR and immune checkpoints is required, before CALR becomes a widely accepted prognostic indicator and therapeutic target for both clinicians and policymakers.

## 4 CONCLUSIONS

We used publicly available gene expression data on pancreatic cancer patients to study the importance of checkpoint ligands, and propose CALR as a more promising target for the treatment of pancreatic cancer. We concluded that checkpoint blockade is a poor option for pancreatic cancer patients based on the expression pattern of checkpoint ligands in pancreatic tumor tissues. Although checkpoint blockade has not shown any significant effect in pancreatic cancer patients, it may be problematic to solely use gene expression patterns to determine whether specific targets are successful or not. Based solely on gene expression data, we propose that targeting CALR is a better option. In addition, while the open source tools are useful for generating hypotheses, care should be taken when conclusions are drawn solely based on gene expression data. The relationship between CALR expression and checkpoint ligands is interesting; however, this must be confirmed by specifically examining how they are co‐regulated in a separate experiment. Thus, the findings of this study present an interesting new hypothesis; however, further biological validation is required to support this conclusion.

## CONFLICT OF INTEREST

The authors declare no conflict of interest.

## Supporting information

Supporting Information.

## Data Availability

All the datasets analyzed during the current study are available in the TCGA. All the data generated during this study are included in the published article. Further information is available from the corresponding author on reasonable request.
