# Supplementary material for: Calreticulin couples with immune checkpoints in pancreatic cancer
Source: Clin Transl Med. 2020 Apr 18;10(1):36–44. doi: 10.1002/ctm2.10 (PMC7239268; doi:10.1002/ctm2.10)
Supplement: Supplementary file 1 — Supporting Information. [file CTM2-10-36-s001.pdf]

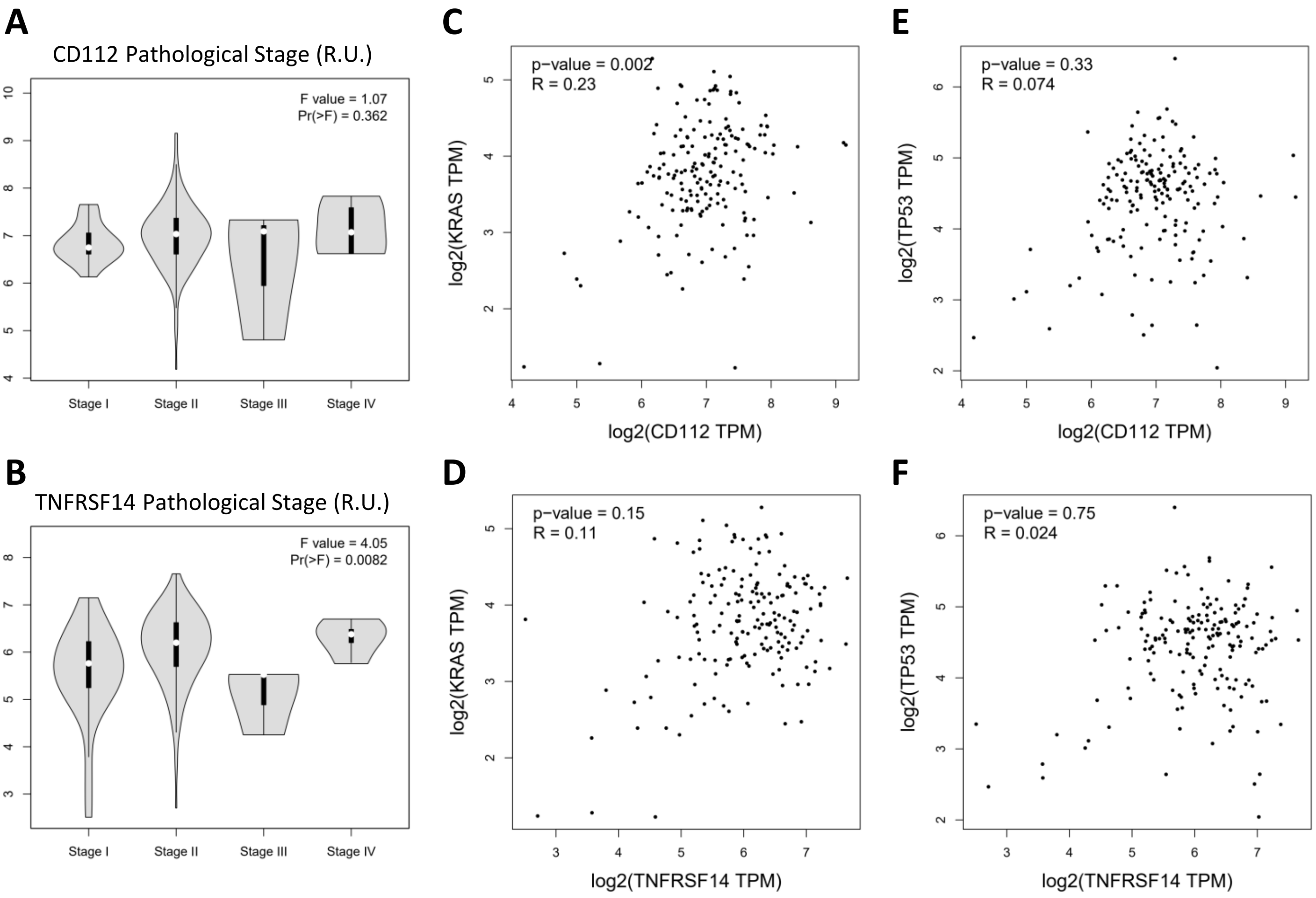

G

Pathological Correlation of Immune Checkpoints

Checkpoint	Co-Stage	F value	Pr(>F)	Co-KRAS	p-Value	R	Co-TP53	p-Value	R
PD-L1	No	0.886	0.449	Yes	2.8e−06	0.34	Yes	1.7e−05	0.32
PD-L2	No	1.03	0.383	Yes	0.033	0.16	Yes	0.00023	0.27
CD80	Yes	3.3	0.0216	Yes	0.0075	0.2	Yes	0.0049	0.21
CD86	No	1.55	0.204	No	0.13	0.11	Yes	0.0013	0.24
VTCN1	Yes	3.53	0.0162	No	0.69	0.03	No	0.47	0.055
VSIR	No	2.03	0.111	Yes	0.00071	0.25	Yes	0.0072	0.2
HHLA2	No	2.16	0.0943	Yes	4.8e−09	0.42	No	0.13	0.11
TNFRSF14	Yes	4.05	0.0082	No	0.15	0.11	No	0.75	0.024
PVR	No	1.5	0.216	Yes	8.4e−09	0.41	No	0.44	0.058
CD112	No	1.07	0.362	Yes	0.002	0.23	No	0.33	0.074
CD200	Yes	4.4	0.00521	No	0.82	−0.017	No	0.15	0.11
LGALS9	Yes	6.83	0.000224	Yes	7.7e−05	0.29	Yes	0.0042	0.21
ICOSLG	No	1.65	0.18	Yes	0.00015	0.28	Yes	0.00028	0.27
TNFSF9	No	1.67	0.176	Yes	0.008	0.2	Yes	0.032	0.16
TNFSF4	No	2.58	0.0552	Yes	6.8e−05	0.29	No	0.18	0.1
CD70	No	2.46	0.0646	No	0.22	0.092	Yes	0.036	0.16
TNFSF18	No	0.727	0.537	Yes	0.021	0.17	No	0.21	0.095
CD48	No	1.01	0.39	No	0.19	−0.098	Yes	0.00068	0.25

Figure S1

A

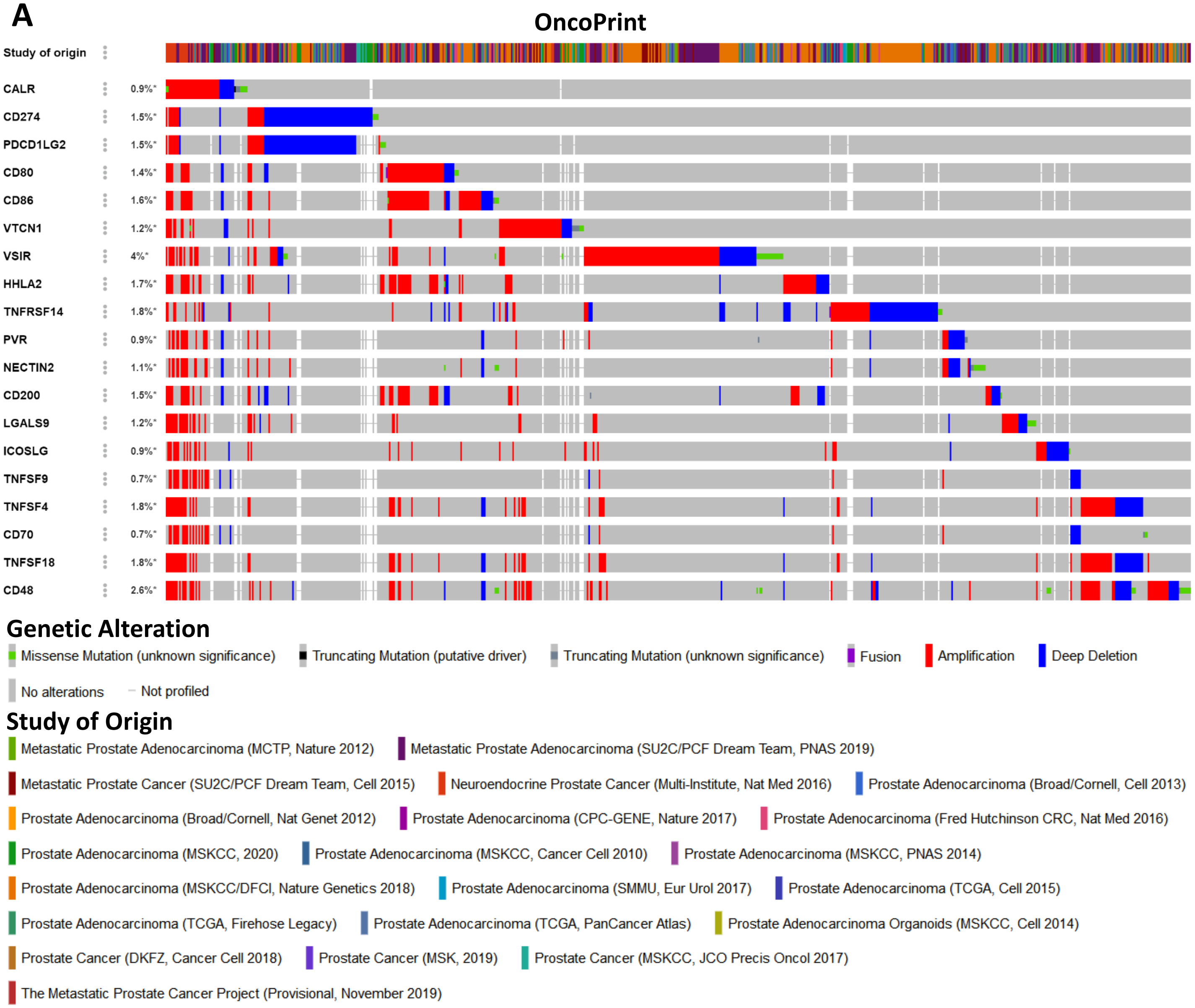

B

Mutual Exclusivity

A	B	Neither	A Not B	B Not A	Both	Log2 Odds Ratio	p-Value	q-Value	Tendency	Significant
CALR	TNFSF9	4328	31	11	22	>3	<0.001	<0.001	Co-occurrence	Yes
CALR	CD70	4325	32	14	21	>3	<0.001	<0.001	Co-occurrence	Yes
CALR	LGALS9	4301	31	38	22	>3	<0.001	<0.001	Co-occurrence	Yes
CALR	PVR	4312	37	27	16	>3	<0.001	<0.001	Co-occurrence	Yes
CALR	NECTIN2	4302	37	37	16	>3	<0.001	<0.001	Co-occurrence	Yes
CALR	ICOSLG	5990	44	41	15	>3	<0.001	<0.001	Co-occurrence	Yes
CALR	TNFSF18	4265	35	74	18	>3	<0.001	<0.001	Co-occurrence	Yes
CALR	TNFSF4	4263	35	76	18	>3	<0.001	<0.001	Co-occurrence	Yes
CALR	CD48	4225	33	114	20	>3	<0.001	<0.001	Co-occurrence	Yes
CALR	HHLA2	4270	36	69	17	>3	<0.001	<0.001	Co-occurrence	Yes
CALR	CD200	4280	37	59	16	>3	<0.001	<0.001	Co-occurrence	Yes
CALR	CD86	4270	37	69	16	>3	<0.001	<0.001	Co-occurrence	Yes
CALR	VTCN1	5968	46	63	13	>3	<0.001	<0.001	Co-occurrence	Yes
CALR	CD80	4273	39	66	14	>3	<0.001	<0.001	Co-occurrence	Yes
CALR	CD274	5948	47	83	12	>3	<0.001	<0.001	Co-occurrence	Yes
CALR	PDCD1LG2	5086	44	77	12	>3	<0.001	<0.001	Co-occurrence	Yes
CALR	VSIR	4172	37	167	16	>3	<0.001	<0.001	Co-occurrence	Yes
CALR	TNFRSF14	5927	48	104	11	>3	<0.001	<0.001	Co-occurrence	Yes

Figure S2

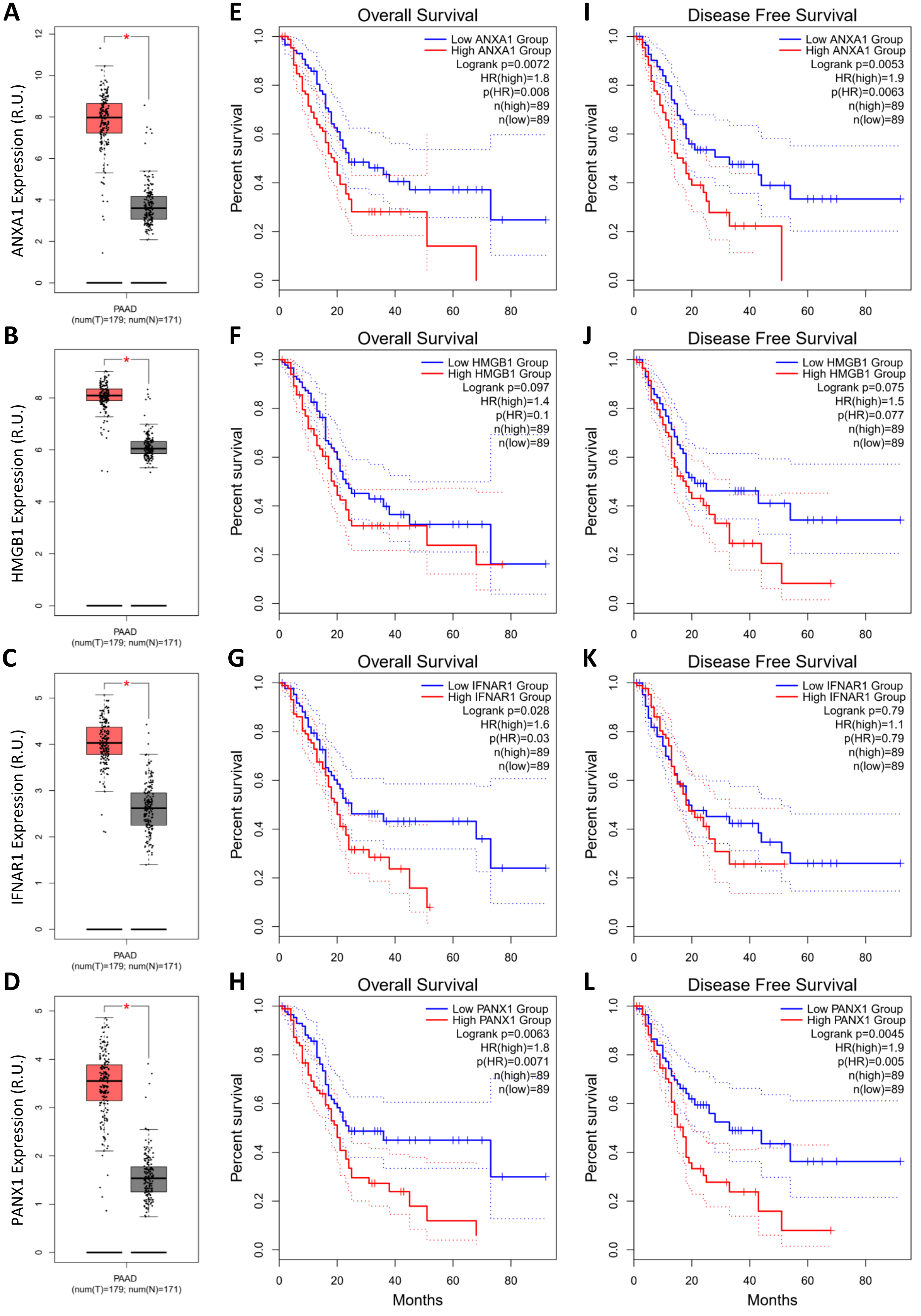

A
ANXA1 Expression (R.U.)
PAAD
(num(T)=179; num(N)=171)
B
HMGB1 Expression (R.U.)
PAAD
(num(T)=179; num(N)=171)
C
IFNAR1 Expression (R.U.)
PAAD
(num(T)=179; num(N)=171)
D
PANX1 Expression (R.U.)
PAAD
(num(T)=179; num(N)=171)
E
Overall Survival
Low ANXA1 Group
High ANXA1 Group
Logrank p=0.0072
HR(high)=1.8
p(HR)=0.008
n(high)=89
n(low)=89
F
Overall Survival
Low HMGB1 Group
High HMGB1 Group
Logrank p=0.097
HR(high)=1.4
p(HR)=0.1
n(high)=89
n(low)=89
G
Overall Survival
Low IFNAR1 Group
High IFNAR1 Group
Logrank p=0.028
HR(high)=1.6
p(HR)=0.03
n(high)=89
n(low)=89
H
Overall Survival
Low PANX1 Group
High PANX1 Group
Logrank p=0.0063
HR(high)=1.8
p(HR)=0.0071
n(high)=89
n(low)=89
I
Disease Free Survival
Low ANXA1 Group
High ANXA1 Group
Logrank p=0.0053
HR(high)=1.9
p(HR)=0.0063
n(high)=89
n(low)=89
J
Disease Free Survival
Low HMGB1 Group
High HMGB1 Group
Logrank p=0.075
HR(high)=1.5
p(HR)=0.077
n(high)=89
n(low)=89
K
Disease Free Survival
Low IFNAR1 Group
High IFNAR1 Group
Logrank p=0.79
HR(high)=1.1
p(HR)=0.79
n(high)=89
n(low)=89
L
Disease Free Survival
Low PANX1 Group
High PANX1 Group
Logrank p=0.0045
HR(high)=1.9
p(HR)=0.005
n(high)=89
n(low)=89
Percent survival
Months

Figure S3

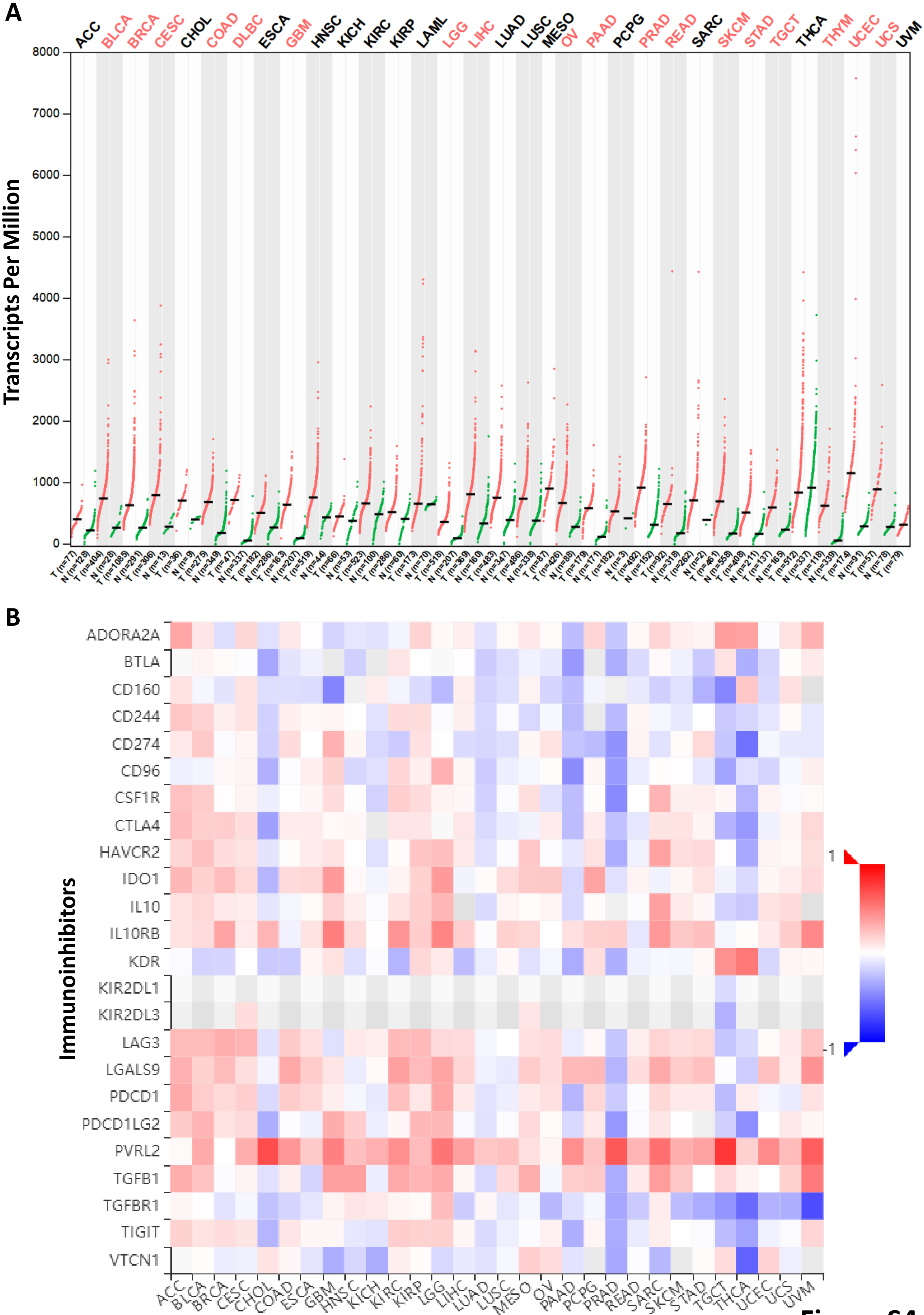
A
ACC BLCA BRCA CESC CHOL COAD DLBC ESCA GBM HNSC KICH KIRC KIRP LAML LGG LIHC LUAD LUSC MESO OV PAAD PCPG PRAD READ SARC SKCM STAD TGCT THCA THYM UCEC UCS UVM
Transcripts Per Million
8000
7000
6000
5000
4000
3000
2000
1000
0
B
ADORA2A
BTLA
CD160
CD244
CD274
CD96
CSF1R
CTLA4
HAVCR2
IDO1
IL10
IL10RB
KDR
KIR2DL1
KIR2DL3
LAG3
LGALS9
PDCD1
PDCD1LG2
PVRL2
TGFB1
TGFBR1
TIGIT
VTCN1
Immunoinhibitors
1
-1
ACC BLCA BRCA CESC CHOL COAD ESCA GBM HNSC KICH KIRC KIRP LGG LIHC LUAD LUSC MESO OV PAAD PCPG PRAD READ SARC SKCM STAD TGCT THCA UCEC UCS UVM

Figure S4
